# Design and construction of artificial metabolic pathways for the bioproduction of useful compounds

**DOI:** 10.5511/plantbiotechnology.24.0721c

**Published:** 2024-09-25

**Authors:** Tomokazu Shirai

**Affiliations:** 1 RIKEN Center for Sustainable Resource Science, Cell Factory Research Team, 1-7-22 Suehiro-cho, Tsurumi-ku, Yokohama, Kanagawa 230-0045, Japan

**Keywords:** artificial metabolic pathway, flux balance analysis, genome-scale metabolic model, plant synthetic biology

## Abstract

To efficiently produce useful compounds using biological cells, it is essential to optimally design all metabolic reactions and pathways, including not only the flow of carbon within the cell but also the production and consumption of energy and the balance of oxidation-reduction. Computational scientific methods are effective for the rational design of metabolic pathways and the optimization of metabolic fluxes. Based on this blueprint, it is crucial to accurately construct the cell, test and analyze whether it conforms to the design, and learn from the results to redesign the system in an effective cycle. This review introduces essential metabolic design techniques in synthetic biology and discusses the potential of using plant cells or plant genes effectively in synthetic biology for the production of useful compounds.

## Introduction

In the biotechnology industry, traditional microbial “fermentation” has been widely used for the production of useful compounds. In recent years, the production of useful compounds through microbial “fermentation” has been employed as one of the techniques for utilizing non-fossil raw materials. This involves using sugars, fixed as carbonates by plants or photosynthetic microorganisms, as carbon sources and producing desired useful compounds through genetically modified microbes. The United States and Europe lead in this “synthetic biology” technology for microbial production, and many common chemical products are already cataloged for production by fermentation. Specific examples of cataloged common chemical products include biofuels such as bioethanol (Enquist-Newman et al. 2013; Jung et al. 2015; Pereira et al. 2010; Yamada et al. 2017) and biobutanol (Chin et al. 2017; Shen et al. 2011), lactic acid which is a raw material for polylactic acid (Liu et al. 2023; Wakai et al. 2014), as well as 1,3-propanediol (Lee et al. 2018; Wilkens et al. 2012), γ-aminobutyric acid (Jorge et al. 2017; Wei et al. 2022; Zhang et al. 2022), and 4-aminocinnamic acid (Minakawa et al. 2019), which are common polymer materials. Furthermore, in the manufacturing industry, the U.S. biotech venture Genomatica and the German company BASF have jointly succeeded in producing tens of thousands of tons per year of 1,4-butanediol (BDO), a key common compound. (Burgard et al. 2016). However, all of these productions use plant biomass as the carbon source, necessitating the conversion of carbon fixed by plants into sugars, which are then stored in the form of polymers such as cellulose, starch, or glycogen. The biomass then needs to be broken down again, either chemically or biologically, to obtain glucose, xylose, and other components, a process that requires a significant amount of energy. Therefore, biomanufacturing using plant cells in synthetic biology is expected. The establishment of plant synthetic biology (PSB) technology that directly utilizes carbon dioxide as a carbon source, metabolizing it to produce target compounds while using it as a nutrient source for building their own cells, is desired.

When producing useful compounds in biological cells, it is essential to optimally design all “metabolic” reactions, including the flow of carbon within the cell, energy production and consumption, and the balance of oxidation-reduction. Especially in recent years, rapid annotation enabled by innovations in genome sequencing technology and information processing technology has made it possible to describe all metabolic reactions on a computer at the genome scale (genome-scale metabolic model: GSM) (Domenzain et al. 2022; Gu et al. 2019). Using GSM, it is now possible to computationally optimize all intracellular metabolic reactions for efficient production of the target compound. Based on the designed metabolic pathways, cells are precisely constructed, and actual experiments are conducted to test whether they conform to the blueprint. Advanced methods, including omics analysis, are used to analyze intracellular metabolism, and the results are learned from to redesign the cycle. This Design-Build-Test-Learn cycle, known as the DBTL cycle, has become systematized and functions effectively, enabling the construction of targeted cells at high throughput, particularly when combined with robotics and automation technologies (Hillson et al. 2019). In this sense, the initial rational metabolic design technique is indispensable, and tuning the metabolic flux using GSMs is a critical step. However, existing GSMs primarily list metabolic reactions of the host cell only. Therefore, if other species have a metabolic reaction that the host cell does not, it is not possible to design the metabolism for the production of the target compound using existing GSMs. Moreover, efficient metabolic design using metabolic reactions from non-host cells, like bypass pathways, is challenging. Another significant limitation of existing design tools based on GSM is that they are essentially limited to naturally occurring compounds in the body. Therefore, the pathways and genes that can be used for the production of the target compound are limited. This review introduces essential metabolic design techniques in synthetic biology and discusses the challenges and possibilities of bioproduction of useful compounds through synthetic biology using plant cells or plant genes.

## Flux Balance Analysis-based metabolic design

Computational scientific methods using linear programming have made it possible to predict rational intracellular metabolic reaction quantities for the bioproduction of target compounds. This is referred to as Flux Balance Analysis (FBA) (Orth et al. 2010). Currently, research is actively conducted to systematically carry out metabolic design through FBA using GSMs, followed by validation through actual experiments, thus enhancing the productivity of target compounds at high throughput. Specifically, this involves constructing a metabolic network based on the GSM, mathematically representing chemical reactions and substance transport occurring within the cells. By balancing all the chemical reaction fluxes within the metabolic network, it is possible to analyze the dynamics of substance metabolism. FBA is particularly useful for understanding the function of biological metabolic pathways and physiological phenomena. For example, to identify nutrients that a microorganism can use as a carbon source, one can construct the metabolic network of that microorganism and perform FBA. Additionally, FBA can be used to analyze and predict abnormal fluxes for elucidating metabolic abnormalities that cause diseases. In FBA, equations like the following are used to balance the fluxes of chemical reactions. 
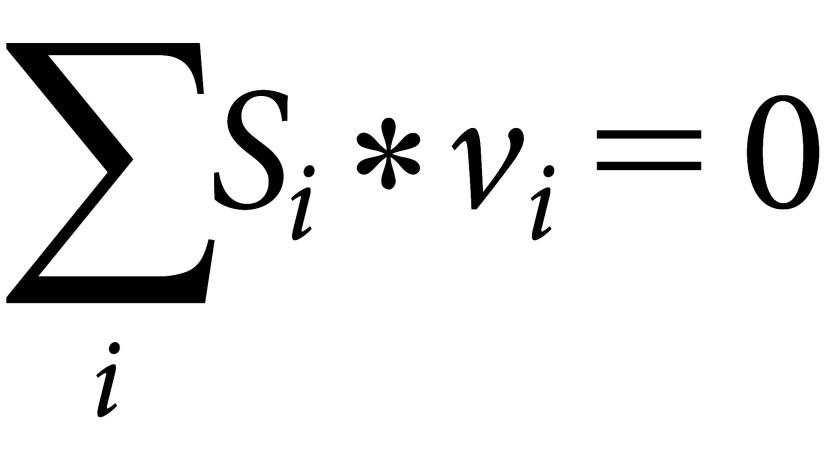


Here, *S_i_* represents the matrix of coefficients for reactants and products in the *i^th^* chemical reaction, and *v_i_* indicates the flux (reaction rate) of the *i^th^* reaction. This equation implies balancing the total flux of all reactions so that their sum equals zero. The equation is linear and can be mathematically solved. Specifically, if we denote the coefficient matrix composed of *S_i_* as *A* and the vector containing *v_i_* as *x*, the above equation can be written as *Ax*=**0**. By setting the production flux of the target compound or the cell growth rate as the objective function, a solution can be obtained using linear programming. When aiming for high production and yield of a target compound, optimization of all intracellular metabolic reactions and enhancement or knockout of relevant genes are essential, but these targets are often multiple. With at least 1,000 metabolic reactions in a cell, considering their comprehensive combinations leads to a computational explosion, making it realistically impossible. For example, even the combination of just two reactions involves about 500,000 possibilities (_1000_C_2_). To solve this problem, various effective computational tools have been developed. OptKnock is a powerful algorithm that identifies and subsequently removes metabolic reactions that are capable of coupling cellular growth with chemical production (Burgard et al. 2003). The FastPros algorithm, which introduces the concept of shadow pricing, excludes metabolic reactions that are not expected to affect the production of the target compound from the search, thus enabling the identification of effective combinations of more than 10 metabolic reaction deletions when paired with the OptKnock algorithm (Ohno et al. 2014). Furthermore, the algorithm has been developed that simply repeat the use of the basic single-level linear programing to extract candidates for reaction deficiencies that would lead to high production of the target compound (Shirai and Kondo 2022). As the algorithm significantly reduce computational burden, they are expected to be extremely useful for metabolic design in bioproduction using plant cells, which have a vast number of metabolic reactions, compared to microbial cells. Basically, these are algorithms that explore combinations of necessary knockout reactions while coupling cell growth with the production of the target compound.

Currently, 41 types of genome-scale metabolic models (GSMs) for plants are available in the Model SEED database (https://modelseed.org/ (Accessed Jan 1, 2024)). These models do not encompass the complete metabolism of each plant, but are limited to modeling certain metabolic pathways or systems related to the plant’s phenotype (see Table 1). Among these, *Arabidopsis thaliana*, one of the most representative plant models, has four developed GSMs. Notably, the GSM developed by Cheung et al. accurately predicts the metabolic fluxes centered on the glycolysis pathway during glucose assimilation (Cheung et al. 2013). Unlike heterotrophic microorganisms, when designing metabolism using plants with carbon dioxide as the sole carbon source, experimental data on the amount of photons, which can serve as reducing power, greatly affects the accuracy of the results. If it is not possible to measure the photon amount, it is necessary to predict the consumption of photons by plant cells under those conditions using as many other phenotypic data as possible, such as growth rate and the production rate of metabolites.

**Table 1:** Table 1. Current genome-scale metabolic models of plants and their applications.

Model name	Species	Application	Reference
Arameta	*Arabidopsis thaliana*	The production of all *A. thaliana* biomass components.	Poolman et al. 2009
AraGEM	*Arabidopsis thaliana*	Successfully simulation of plant metabolic function of photorespiration and respiration.	de Oliveira Dal’Molin et al. 2010a
Aramodel2012	*Arabidopsis thaliana*	Study for up to ten submodels for specific tissue types and growth stages in isolation.	Mintz-Oron et al. 2012
Aracell	*Arabidopsis thaliana*	Flux prediction of heterotrophic Arabidopsis cells in glucose assimilation under some conditions.	Cheung et al. 2013
iRS1563	*Zea mays*	Lignin biosynthesis and the effects of changes in cell wall composition based on updated version of AraGEM.	Saha et al. 2011
C4GEM	*Sorghum bicolor*	Multiple cell type interaction accessing C4 photosynthesis in mesophyll and bundle sheath cells.	de Oliveira Dal’Molin et al. 2010b
iSM1809	*Nothapodytes nimmoniana*	Identify and rank suitable reaction targets for overexpression and knockout for camptothecin production.	Murali et al. 2023
—	*Glycine max*	Investigation of the mobilization during seedling growth and adaptation of the model to reflect metabolism in the cotyledons and hypocotyl/ root axis.	Moreira et al. 2019
3.5v5 Medicago	*Medicago truncatula*	Investigation of the effects of the symbiosis on metabolic fluxes and plant growth by connecting this model to a model of its rhizobial symbiont, *Sinorhizobium meliloti.*	Pfau et al. 2018
iEC7871	*Quercus suber*	The tissue-specific models to predict interactions among the three tissues at the light and dark phases with transcriptomics data, which is used to analyze the synthesis of suberin monomers production.	Cunha et al. 2023

## Design incorporating metabolic pathways derived from different organisms

In FBA using existing GSMs, it is difficult to design efficient metabolism utilizing metabolic reactions from non-host cells. Introducing pathways outside the host cell’s own, such as bypass routes, can enable more efficient metabolic designs with higher yields. For instance, there are many examples like the introduction of the glyoxylate pathway during succinic acid production in glycogen dissimilation metabolism using cyanobacteria (Durall et al. 2021), lysine production using NADP-dependent glyceraldehyde 3-phosphate dehydrogenase (GapN) (Takeno et al. 2010), increased flux of Acetyl-CoA production by using bypass routes other than pyruvate dehydrogenase in the glycolysis (Ku et al. 2020), and efficient material production through the introduction of parallel metabolic pathways during simultaneous metabolism of glucose and xylose (Fujiwara et al. 2020). Although these efficient metabolic pathway introductions require the experience and insights of many researchers, they can be time-consuming when aiming for the efficient production of many useful compounds in the future. Tools that add metabolic reactions and pathways from information databases like KEGG (http://www.genome.jp/kegg/ (Accessed Jan 1, 2024)) or BRENDA (http://www.brenda-enzymes.org (Accessed Jan 1, 2024)) to host reactions on the computer, aiming for high-yield production of target compounds, are considered very effective (Blaß et al. 2017; Shirai et al. 2016). For this, the databasing of metabolic reactions and corresponding gene information in plant cells is essential and expected to contribute significantly to expanding bio-compound production, which has primarily been done in microbial hosts, to plant cells.

## Design of artificial metabolic pathways

One of the major limitations of existing design tools based on FBA using GSMs is that they are essentially bound to the structures of compounds that naturally exist within living organisms. As a result, the reaction pathways and enzymes available for the production of the target compound are also limited. To solve such issues, some tools have been developed that teach computers the enzyme reaction patterns found in databases like KEGG and BRENDA, enabling the design of novel metabolic reactions, or artificial metabolic reactions (Araki et al. 2015; Kumar et al. 2018; Kuwahara et al. 2016). If these designed artificial metabolic reactions, starting with known biological compounds, can be realized, it becomes possible to biosynthesize the target compound. Based on these designs, biosynthetic pathways are constructed in actual experiments. Specifically, this involves introducing mutations into natural enzymes found in nature to alter their substrate specificity and enhance their activity, thereby acquiring the desired metabolic pathways (Mori and Shirai 2018). In fact, there are known cases of successful biosynthesis of useful chemicals derived from fossil resources, which were not previously known to be produced by biological organisms (Luo et al. 2023; Mori et al. 2021). In plant cells, a far greater number of metabolic products are biosynthesized compared to microbes, and many useful compounds have historically been produced through the extraction from plants. If plant synthetic biology (PSB) could be used to industrially produce target compounds through fermentation methods, it would be beneficial. However, there are often situations where the metabolic pathways are complex, and the biosynthetic pathways are unclear, or parts of the synthesis pathways remain unknown. Tools mentioned above could be effective in predicting these metabolic pathways, including the missing links. If biosynthetic pathways can be predicted, it may become possible to identify functioning plant genes based on the corresponding enzyme reaction patterns and annotation information. Alternatively, entirely different biosynthetic pathways could be predicted and constructed within plant cells, potentially greatly improving the production efficiency of target compounds that have been inefficient until now.

## Potential and challenges of synthetic biology using plant cells

In the production of useful compounds through synthetic biology, microbial cells have primarily been used as hosts to date. They offer numerous advantages, such as a wealth of genetic modification tools for breeding, rapid growth, and a variety of cultivation conditions. However, microbes fundamentally require a supply of sugar, necessitating the breakdown of polymers of sugar stored by plants. In contrast, plant cells can directly fix carbon dioxide, contributing directly to carbon negativity. Therefore, the establishment of technology for one-stop production of target compounds from carbon dioxide using plant cells is desirable. A major bottleneck in using plant cells is their relatively low carbon dioxide assimilation capacity, especially when considering industrial-scale production of target compounds. For example, the glucose consumption rate of *Escherichia coli* is 2–10 mmol g^−1^-DCW h^−1^ (Fragoso-Jiménez et al. 2022), which translates to 120–600 mmol-carbon g^−1^-DCW h^−1^. In contrast, plant cells have a growth rate of about 0.02 h^−1^ (Ryu et al. 1990), indicating that their carbon dioxide assimilation rate is only a few millimoles g^−1^-DCW h^−1^. First and foremost, it is essential to significantly increase the carbon dioxide uptake rate and enhance growth rates. While RuBisCo in plant cells is well-known for its carbon dioxide uptake function, introducing alternative pathways for carbon dioxide uptake or constructing new artificial metabolic pathways could expand the potential of synthetic biology in plant cells. The issue of slow growth and production rates due to the low rate of carbon dioxide uptake by RuBisCO might be overcome by enhancing or introducing alternative pathways. For example, besides phosphoenolpyruvate carboxylase (EC 4.1.1.31), which is known to be effective in carbon dioxide fixation in C4 plants, pyruvate carboxylase (EC 6.4.1.1), glycine dehydrogenase (EC 1.4.4.2), and formate dehydrogenase (EC 1.17.1.9) can be considered. It is unclear why plants have not evolved to utilize these carbon dioxide fixation capabilities as standard. However, in the current Earth’s atmosphere where oxygen concentration is 500 times that of carbon dioxide, RuBisCO can be considered a rational enzyme from the viewpoints of enzyme quantity and substrate affinity. Nevertheless, in the creation of useful plant using synthetic biology, it may be necessary to add new carbon fixation capabilities other than RuBisCO to increase productivity. Simultaneously, as Lin et al. (2014) have shown, engineering RuBisCO with a carbon dioxide-concentrating mechanism is expected to achieve more efficient carbon fixation capabilities. Once that is achieved, the metabolic design methods and tools introduced earlier, as well as optimization of intracellular metabolism using GSM, become crucial steps and can provide powerful assistance. When aiming for bioproduction of high-value pharmaceuticals or useful compounds derived from fossil resources, these compounds or their intermediate metabolites could be toxic to microbial cells. Plants, compared to microbes, can synthesize a much wider range of metabolic products and have strong resistance to various stresses, including toxicity. Since plant cells have multiple compartments other than the cytoplasm, it is possible to construct pathways in compartments like peroxisomes to convert toxic compounds into less harmful ones before moving them to the cytoplasm for final production. This compartmentalized approach can also be theoretically applied in eukaryotic microbes like yeast, although they cannot directly utilize carbon dioxide as a nutrient source and have a narrower variety of synthesizable compounds compared to plants. Considering direct production of various useful compounds from carbon dioxide, synthetic biology using plant cells offers advantages. Advancing plant synthetic biology is not only beneficial for manufacturing with plant cells but could also contribute to improving carbon dioxide fixation rates, potentially leading to significant advances in direct carbon dioxide fixation by microbes like *E. coli* and yeast. Furthermore, as artificial synthetic pathways for useful compounds are constructed and intracellular metabolism is optimized in plant cells, valuable genes and enzymes will be accumulated. For certain bioproducts produced by microbes, enzymes or genes derived from plants are already being used. However, due to the limited number of genes available in databases, introducing these genes into *E. coli* or yeast often results in insufficient expression or no expression at all. Even if expression is achieved, incomplete translation or insolubilization of proteins, rendering them non-functional as enzymes, is a real possibility. Advancing synthetic biology in plant cells requires building databases focused on bio-compound production. By progressing in parallel, the establishment of synthetic biological engineering using plant cells can be expected.

## Perspective

The construction of artificial metabolic pathways through the metabolic design techniques discussed thus far enables the direct bioproduction of useful compounds derived from fossil resources (such as fuels, common plastics, and engineering plastics). Traditionally, compounds that are easily biosynthesized by organisms have been produced through fermentation, then refined and chemically transformed to obtain the final products. This approach often required the input of a large amount of energy for the total production process, which in many cases hindered practical application and commercialization. The construction of artificial metabolic pathways often requires genes derived from plants, and establishing plant synthetic biology (PSB) is also crucial for eliminating the effects of toxicity of the product or its intermediates. In particular, the useful compounds obtained through PSB are the same as those obtained through oil refineries, making it relatively easy to integrate them with existing infrastructure, such as chemical complexes, and facilitating research that fuses chemistry and biology for practical application in process development. Currently, as society demands the achievement of the Sustainable Development Goals (SDGs) and the global progression towards a decarbonized society and circular economy, the production of useful compounds using plant cells through synthetic biology can be a powerful means to achieve carbon negativity. Therefore, PSB is considered to directly contribute to these objectives.

## Abbreviations

BDO: 1,4-butanediol
BRENDA: Braunschweig Enzyme Database
DBTL cycle: Design-Build-Test-Learn cycle
FBA: flux balance analysis
GSM: genome-scale metabolic model
KEGG: Kyoto Encyclopedia of Genes and Genomes
PSB: plant synthetic biology
SDGs: Sustainable Development Goals

## References

[RAraki2015] Araki M, Cox RS 3rd, Makiguchi H, Ogawa T, Taniguchi T, Miyaoku K, Nakatsui M, Hara KY, Kondo A (2015) M-path: A compass for navigating potential metabolic pathways. *Bioinformatics* 31: 905–911 25398612 10.1093/bioinformatics/btu750

[d67e528] Blaß LK, Weyler C, Heinzle E (2017) Network design and analysis for multi-enzyme biocatalysis. *BMC Bioinformatics* 18: 366 28797226 10.1186/s12859-017-1773-y PMC5553788

[RBurgard2016] Burgard A, Burk MJ, Osterhout R, Dien SV, Yim H (2016) Development of a commercial scale process for production of 1,4-butanediol from sugar. *Curr Opin Biotechnol* 42: 118–125 27132123 10.1016/j.copbio.2016.04.016

[RBurgard2003] Burgard AP, Pharkya P, Maranas CD (2003) Optknock: A bilevel programming framework for identifying gene knockout strategies for microbial strain optimization. *Biotechnol Bioeng* 84: 647–657 14595777 10.1002/bit.10803

[RCheung2013] Cheung CYM, Williams TCR, Poolman MG, Fell DA, Ratcliffe RG, Sweetlove LJ (2013) A method for accounting for maintenance costs in flux balance analysis improves the prediction of plant cell metabolic phenotypes under stress conditions. *Plant J* 75: 1050–1061 23738527 10.1111/tpj.12252

[RChin2017] Chin WC, Lin KH, Liu CC, Tsuge K, Huang CC (2017) Improved n-butanol production via co-expression of membrane-targeted tilapia metallothionein and the clostridial metabolic pathway in *Escherichia coli.* *BMC Biotechnol* 17: 36 28399854 10.1186/s12896-017-0356-3 PMC5387206

[RCunha2023] Cunha E, Silva M, Chaves I, Demirci H, Lagoa DR, Lima D, Rocha M, Rocha I, Dias O (2023) The first multi-tissue genome-scale metabolic model of a woody plant highlights suberin biosynthesis pathways in *Quercus suber.* *PLoS Comput Biol* 19: e1011499 37729340 10.1371/journal.pcbi.1011499 PMC10545120

[Rde2010a] de Oliveira Dal’Molin CG, Quek LE, Palfreyman RW, Brumbley LK, Nielsen LK (2010a) AraGEM, a genome-scale reconstruction of the primary metabolic network in *Arabidopsis.* *Plant Physiol* 152: 579–589 20044452 10.1104/pp.109.148817 PMC2815881

[Rde2010b] de Oliveira Dal’Molin CG, Quek LE, Palfreyman RW, Brumbley LK, Nielsen LK (2010b) C4GEM, a genome-scale metabolic model to study C4 plant metabolism. *Plant Physiol* 154: 1871–1885 20974891 10.1104/pp.110.166488 PMC2996019

[RDomenzain2022] Domenzain I, Sánchez B, Anton M, Kerkhoven EJ, Millán-Oropeza A, Henry C, Siewers V, Morrissey JP, Sonnenschein N, Nielsen J (2022) Reconstruction of a catalogue of genome-scale metabolic models with enzymatic constraints using GECKO 2.0. *Nat Commun* 13: 3766 35773252 10.1038/s41467-022-31421-1 PMC9246944

[RDurall2021] Durall C, Kukil K, Hawkes JA, Albergati A, Lindblad P, Lindberg P (2021) Production of succinate by engineered strains of *Synechocystis* PCC 6803 overexpressing phosphoenolpyruvate carboxylase and a glyoxylate shunt. *Microb Cell Fact* 20: 39 33557832 10.1186/s12934-021-01529-y PMC7871529

[REnquist2013] Enquist-Newman M, Faust AM, Bravo DD, Santos CN, Raisner RM, Hanel A, Sarvabhowman P, Le C, Regitsky DD, Cooper SR, et al. (2013) Efficient ethanol production from brown macroalgae sugars by a synthetic yeast platform. *Nature* 505: 239–243 24291791 10.1038/nature12771

[RFragoso2022] Fragoso-Jiménez JC, Gutierrez-Rios RM, Flores N, Martinez A, Lara AR, Delvigne F, Gosset G (2022) Glucose consumption rate-dependent transcriptome profiling of *Escherichia coli* provides insight on performance as microbial factories. *Microb Cell Fact* 21: 189 36100849 10.1186/s12934-022-01909-y PMC9472385

[RFujiwara2020] Fujiwara R, Noda S, Tanaka T, Kondo A (2020) Metabolic engineering of *Escherichia coli* for shikimate pathway derivative production from glucose-xylose co-substrate. *Nat Commun* 11: 279 31937786 10.1038/s41467-019-14024-1 PMC6959354

[RGu2019] Gu C, Kim GB, Kim WJ, Kim HU, Lee SY (2019) Current status and applications of genome-scale metabolic models. *Genome Biol* 20: 121 31196170 10.1186/s13059-019-1730-3 PMC6567666

[RHillson2019] Hillson N, Caddick M, Cai Y, Carrasco JA, Chang MW, Curach NC, Bell DJ, Le Feuvre R, Friedman DC, Fu X, et al. (2019) Building a global alliance of biofoundries. *Nat Commun* 10: 2040 31068573 10.1038/s41467-019-10079-2 PMC6506534

[RJorge2017] Jorge JM, Nguyen AQ, Pérez-García F, Kind S, Wendisch VF (2017) Improved fermentative production of gamma-aminobutyric acid via the putrescine route: Systems metabolic engineering for production from glucose, amino sugars, and xylose. *Biotechnol Bioeng* 114: 862–873 27800627 10.1002/bit.26211

[RJung2015] Jung YH, Park HM, Kim DH, Park YC, Seo JH, Kim KH (2015) Combination of high solids loading pretreatment and ethanol fermentation of whole slurry of pretreated rice straw to obtain high ethanol titers and yields. *Bioresour Technol* 198: 861–866 26461793 10.1016/j.biortech.2015.09.102

[RKu2020] Ku JT, Chen AY, Lan EI (2020) Metabolic engineering design strategies for increasing acetyl-CoA flux. *Metabolites* 10: 166 32340392 10.3390/metabo10040166 PMC7240943

[RKumar2018] Kumar A, Wang L, Ng CY, Maranas CD (2018) Pathway design using de novo steps through uncharted biochemical spaces. *Nat Commun* 9: 184 29330441 10.1038/s41467-017-02362-x PMC5766603

[RKuwahara2016] Kuwahara H, Alazmi M, Cui X, Gao X (2016) MRE: A web tool to suggest foreign enzymes for the biosynthesis pathway design with competing endogenous reactions in mind. *Nucleic Acids Res* 44(W1): W217–W225 27131375 10.1093/nar/gkw342 PMC4987905

[RLee2018] Lee JH, Jung MY, Oh MK (2018) High-yield production of 1,3-propanediol from glycerol by metabolically engineered *Klebsiella pneumoniae.* *Biotechnol Biofuels* 11: 104 29657579 10.1186/s13068-018-1100-5 PMC5890353

[RLin2014] Lin MT, Occhialini A, Andralojc PJ, Parry MAJ, Hanson MR (2014) A faster Rubisco with potential to increase photosynthesis in crops. *Nature* 513: 547–550 25231869 10.1038/nature13776 PMC4176977

[RLiu2023] Liu T, Sun L, Zhang C, Liu Y, Li J, Du G, Lv X, Liu L (2023) Combinatorial metabolic engineering and process optimization enables highly efficient production of L-lactic acid by acid-tolerant *Saccharomyces cerevisiae.* *Bioresour Technol* 379: 129023 37028528 10.1016/j.biortech.2023.129023

[RLuo2023] Luo ZW, Choi KR, Lee SY (2023) Improved terephthalic acid production from p-xylene using metabolically engineered *Pseudomonas putida.* *Metab Eng* 76: 75–86 36693471 10.1016/j.ymben.2023.01.007

[RMinakawa2019] Minakawa H, Masuo S, Kaneko T, Takaya N (2019) Fermentation and purification of microbial monomer 4-amminocinnamic acid to produce ultra-high performance bioplastics. *Process Biochem* 77: 100–105

[RMintz2012] Mintz-Oron S, Meir S, Malitsky S, Ruppin E, Aharoni A, Shlomi T (2012) Reconstruction of *Arabidopsis* metabolic network models accounting for subcellular compartmentalization and tissue-specificity. *Proc Natl Acad Sci USA* 109: 339–344 22184215 10.1073/pnas.1100358109 PMC3252957

[RMoreira2019] Moreira TB, Shaw R, Luo X, Ganguly O, Kim HS, Coelho LGF, Cheung CYM, Williams TCR (2019) A genome-scale metabolic model of soybean (*Glycine max*) highlights metabolic fluxes in seedlings. *Plant Physiol* 180: 1912–1929 31171578 10.1104/pp.19.00122 PMC6670085

[RMori2021] Mori Y, Noda S, Shirai T, Kondo A (2021) Direct 1,3-butadiene biosynthesis in *Escherichia coli* via a tailored ferulic acid decarboxylase mutant. *Nat Commun* 12: 2195 33850144 10.1038/s41467-021-22504-6 PMC8044207

[RMori2018] Mori Y, Shirai T (2018) Designing artificial metabolic pathways, construction of target enzymes, and analysis of their function. *Curr Opin Biotechnol* 54: 41–44 29452926 10.1016/j.copbio.2018.01.021

[RMurali2023] Murali S, Ibrahim M, Rajendran H, Shagun S, Masakapalli SK, Raman K, Srivastava S (2023) Genome-scale metabolic model led engineering of *Nothapodytes nimmoniana* plant cells for high camptothecin production. *Front Plant Sci* 14: 1207218 37600193 10.3389/fpls.2023.1207218 PMC10433906

[ROhno2014] Ohno S, Shimizu H, Furusawa C (2014) FastPros: Screening of reaction knockout strategies for metabolic engineering. *Bioinformatics* 30: 981–987 24257186 10.1093/bioinformatics/btt672 PMC3967105

[ROrth2010] Orth JD, Thiele I, Palsson BØ (2010) What is flux balance analysis? *Nat Biotechnol* 28: 245–248 20212490 10.1038/nbt.1614 PMC3108565

[RPereira2010] Pereira FB, Guimarães PM, Teixeira JA, Domingues L (2010) Optimization of low-cost medium for very high gravity ethanol fermentations by *Saccharomyces cerevisiae* using statistical experimental designs. *Bioresour Technol* 101: 7856–7863 20627715 10.1016/j.biortech.2010.04.082

[RPfau2018] Pfau T, Christian N, Masakapalli SK, Sweetlove LJ, Poolman MG, Ebenhöh O (2018) The intertwined metabolism during symbiotic nitrogen fixation elucidated by metabolic modelling. *Sci Rep* 8: 12504 30131500 10.1038/s41598-018-30884-x PMC6104047

[RPoolman2009] Poolman MG, Matiquet L, Sweetlove LJ, Fell DA (2009) A genome-scale metabolic model of Arabidopsis and some of its properties. *Plant Physiol* 151: 1570–1581 19755544 10.1104/pp.109.141267 PMC2773075

[RRyu1990] Ryu DD, Lee SO, Romani RJ (1990) Determination of growth rate for plant cell cultures: Comparative studies. *Biotechnol Bioeng* 35: 305–311 18592523 10.1002/bit.260350312

[RSaha2011] Saha R, Suthers PF, Maranas CD (2011) *Zea mays* iRS1563: A comprehensive genome-scale metabolic reconstruction of maize metabolism. *PLoS One* 6: e21784 21755001 10.1371/journal.pone.0021784 PMC3131064

[RShen2011] Shen CR, Lan EI, Dekishima Y, Baez A, Cho KM, Liao JC (2011) Driving forces enable high-titer anaerobic 1-butanol synthesis in *Escherichia coli.* *Appl Environ Microbiol* 77: 2905–2915 21398484 10.1128/AEM.03034-10 PMC3126405

[RShirai2022] Shirai T, Kondo A (2022) In silico design strategies for the production of target chemical compounds using iterative single-level linear programming problems. *Biomolecules* 12: 620 35625545 10.3390/biom12050620 PMC9138359

[RShirai2016] Shirai T, Osanai T, Kondo A (2016) Designing intracellular metabolism for production of target compounds by introducing a heterologous metabolic reaction based on a *Synechosystis* sp. 6803 genome-scale model. *Microb Cell Fact* 15: 13 26783098 10.1186/s12934-016-0416-8 PMC4717628

[RTakeno2010] Takeno S, Murata R, Kobayashi R, Mitsuhashi S, Ikeda M (2010) Engineering of *Corynebacterium glutamicum* with an NADPH-generating glycolytic pathway for L-lysine production. *Appl Environ Microbiol* 76: 7154–7160 20851994 10.1128/AEM.01464-10 PMC2976228

[RWakai2014] Wakai S, Yoshie T, Asai-Nakashima N, Yamada R, Ogino C, Tsutsumi H, Hata Y, Kondo A (2014) L-lactic acid production from starch by simultaneous saccharification and fermentation in a genetically engineered *Aspergillus oryzae* pure culture. *Bioresour Technol* 173: 376–383 25314668 10.1016/j.biortech.2014.09.094

[RWei2022] Wei L, Zhao J, Wang Y, Gao J, Du M, Zhang Y, Xu N, Du H, Ju J, Liu Q, et al. (2022) Engineering of *Corynebacterium glutamicum* for high-level γ-aminobutyric acid production from glycerol by dynamic metabolic control. *Metab Eng* 69: 134–146 34856366 10.1016/j.ymben.2021.11.010

[RWilkens2012] Wilkens E, Ringel AK, Hortig D, Willke T, Vorlop KD (2012) High-level production of 1,3-propanediol from crude glycerol by *Clostridium butyricum* AKR102a. *Appl Microbiol Biotechnol* 93: 1057–1063 21972131 10.1007/s00253-011-3595-6

[RYamada2017] Yamada R, Nakashima K, Asai-Nakashima N, Tokuhara W, Ishida N, Katahira S, Kamiya N, Ogino C, Kondo A (2017) Direct ethanol production from ionic liquid-pretreated lignocellulosic biomass by cellulase-displaying yeasts. *Appl Biochem Biotechnol* 182: 229–237 27844339 10.1007/s12010-016-2322-2

[RZhang2022] Zhang L, Yue Y, Wang X, Dai W, Piao C, Yu H (2022) Optimization of fermentation for γ-aminobutyric acid (GABA) production by yeast *Kluyveromyces marxianus* C21 in okara (soybean residue). *Bioprocess Biosyst Eng* 45: 1111–1123 35179639 10.1007/s00449-022-02702-2

